# Quack Advertisements—The Secular and Religious Press

**Published:** 1865-05

**Authors:** 


### EDITORIAL DEPARTMENT.

### QUACK ADVERTISEMENTS—THE SECULAR AND RELIGIOUS PRESS.

The English Medical Journals have been exposing and violently condemning the indecency and dishonesty of the public press in admitting to their columns the obscene advertisements of quacks, and many of the leading journals have in consequence signified their purpose to exclude such matter from their columns. From an editorial article in the *Boston Medical and Surgical Journal* we learn that the immediate cause of this movement was a late exposure in the courts, by a victim of these extortionate quacks who had the moral courage to expose this system of robbery. That any exposure should arouse public opinion and the sensibilities of those having control of the public press, is highly gratifying, and shows that the public are too ignorant of the real objects of these villainous notices, and newspaper publishers too ready to publish whatever pays best, provided the public will tolerate in their issues, these vile, disgusting, immoral advertisements. To bring this subject a little nearer home than London, and talk about the same matter in the State of New York, or even city of Buffalo, would perhaps interest our readers more and come nearer within the scope of our personal observation and influence. Newspaper notices of quacks and quack medicines include the major part of what a great many people know about disease and medicine; the daily paper is the almost exclusive source of instruction which many families possess. They read the local items, and they are in the main truthful records of passing events; some of the telegraphic despatches are official, and to be depended upon; the political opinions of their favorite daily, they adopt and regard them as correct; with something of the same feelings of confidence they also read the advertisements, and rarely mistrust that they are not also "official despatches" representing truly and faithfully the facts therein recorded, and to be relied upon and trusted.

Again into the "local column" it is very customary to smuggle notices of some "celebrated doctor," who will be at such a place at such a time, and who cures all manner of incurable diseases, or some medicine which is capable of the greatest wonders; the advertisement reading like an editorial endorsement, and placed by special arrangement in position to be regarded by many of the unobserving as the opinion and recommendation of the editor. By this means many people who are not naturally very great fools, are very greatly fooled; indeed it is a deliberately planned swindle, and there is scarcely a paper published but is guilty of the corruption. The daily commercial papers may perhaps try to excuse themselves on the plea that they are not responsible for the advertisement; let them then become a medium of communicating to the public all manner of vileness, dishonesty, immorality; all sorts of misrepresentation, foolishness and inconsistency; all that will make their readers know less of truth, and more of error.

If this may be said of the secular press, what shall be said of the religious? A distinguished divine publishes upon one side a scriptural sermon, illustrating the great truths of revelation, or enforcing the duties of honesty, morality and christianity, while upon the opposite page, every absurd, untruthful and immoral misrepresentation conceivable, is presented in attractive style, that is, in large type, but disguised form; even the implements of the most disgusting crimes are placed side by side with the pure doctrines of christianity. When our religious papers and journals advertise burglar's tools, counterfitting dies, assassination instruments, gambling saloons and assignation houses, they will have done no meaner and have become no guiltier than, when presenting to the public "Golden Pills," "Monthly Regulators," or "Receipts for Removal of Obstructions," obtained gratuitously of Rev A. B., by enclosing a stamp, etc., etc. The quack medicine and the quack and itinerant doctor occupy a large space in our public prints, both secular and religious, with the effect to make more people fools, than they make either good or wise. We have often laid aside our favorite religious paper, and said the *New York Independent*, is a curse rather than a blessing—will kill with its advertisements of worthless or injurious medicines more bodies and with its advertised falsehoods and misrepresentations ruin more souls, than can by any possibility be redeemed by its agency. What we have thought of it, is equally true of most others; the evil is becoming a great one, its baleful effects are daily witnessed by the physician in his intercourse with the sick, and any influence which can be brought to bear upon the regeneration of the public press, will be of incalculable good. The medical profession may be in fault in this matter; its influence has not been exerted as it might have been; the people have not yet been instructed in truth to the exclusion of error; perhaps they will forever shut out all knowledge of trnth. It is regarded as unprofessional to place the truth of medicine before the people; it is supposed to be beyond them and that they cannot reach it, even if they would. People intelligent in all other things are profoundly ignorant in medicine; and it is mainly on this account that the untruthful and absurd misrepresentations of medicine-mongers exert so great influence over the public.

Publishers should be held in some degree responsible for what they advertise, and *above* all, they should not allow imposture to be extended by appearing as supposed endorsers. The most ready way to correct this great evil would appear to be in educating the people to shun and despise these advertised quacks and medicines, and to avoid the sheets which contain them. This is not an easy task, since the people are ready to pay for being fooled, but would resist being instructed in the truth; these "*Testimonials of Cures*" are regarded as positive evidence and worth any amount of negative testimony. How to correct these notions—how to reach the public ear and instruct the masses in any rational and truthful views of disease and its cure, is truly a question not yet satisfactorily answered. By what medium the public can be reached and this gigantic quack advertisement swindle corrected, we are not prepared to say, but we think the field is one which every physician should cultivate to the extent of his ability; it is "missionary ground"—the greatest field for benevolent effort and open to all honest workers.

All effort in the direction of reform, will of course be opposed by publishers upon the ground that those who pay for the advertising space, should have the privilege of filling it with what they please, as they would say, "furnishing the copy;" by the great public of quacks, who are ever jealous of any attempt at regeneration, upon the claim of the right of free trade, free speech, free thought, and the inalienable privilege of every American man, woman and child, to swallow whatever they please, if advertised as good for them. This opposition, however, is not excuse; we are to go as the apostles of science and preach the gospel of medical truth to those who are capable of receiving it—who are able to understand and appreciate its plainest laws. Alas! it will not be accepted; there are scoffers in these days who will deny its truths, and will persuade others to follow their teachings; but nevertheless "truth is God-like and must finally prevail."

It would seem that there might be found some short way to accomplish for truth a great triumph, and that an intelligent people might be taught a short hand method of reformation; that papers and journals might be silenced in their teachings of immorality, inconsistency, falsehood and indecency; but whoever has watched the current of popular credulity, has discovered that nothing can be invented too preposterous and absurd for belief and adoption by a portion of every community; whoever is acquainted with the *status* of morality, knows that even many in the higher classes—the fashionable circles, are not above the meanest and most contemptable crimes—that they contribute to evils and wrongs, which are covered, yet murderous. It is not easy to make short hand disposal of systems which receive such support and sanction.

Silence in regard to this system of imposition is what it requires for life; with exposure it will die away and disappear; it is only to be known to be shunned. Even those criminally disposed, will shun and despise a fraud so palpable and plain, which, offering them the means of crime, robs them of their money, and disappoints them at last.

Tell many families that advertised doctors and medicines are valueless; that they never do good, and very often harm, and they foolishly suppose that you are prejudiced, and prefer that you call a physician, if in pain, and take his advice, when Perry Davis' Pain Killer would relieve them as early, and cost them much less; but as we have said, "truth is powerful and must prevail." If we would correct the immeasurable swindle of newspaper advertising of quackery, we must speak boldly, and through the press; the people are to be reached in this way, and in no other. If this subject was truthfully written upon and exposed in the same sheet with the abominable nuisance, it would in time abate the nuisance. It cannot be possible that any great number of people would persistently follow error when carefully pointed out; though it must be confessed that about one-fourth of the population have little mental power to distinguish the most palpable error in medicine from the plainest truth.

